# Supplementary material for: Effects of Perioperative Oral Management in Patients with Cancer
Source: J Clin Med. 2022 Nov 6;11(21):6576. doi: 10.3390/jcm11216576 (PMC9655039; doi:10.3390/jcm11216576)
Supplement: Supplementary file 1 [file jcm-11-06576-s001.zip › jcm-1980181-supplementary.pdf]

Journal of
Clinical Medicine

MDPI

Supplementary Table S1. Primary lesions of the No POM and POM groups.

No POM (n=8,208)			POM (n=1,233)		
	n	%		n	%
Breast cancer	1,033	12.6	Colorectal cancer	410	33.3
Stomach cancer	982	12.0	Stomach cancer	303	24.6
Hematopoietic tumor	958	11.7	Lung cancer	175	14.2
Gynecologic cancer	930	11.3	Head and neck cancer	73	5.9
Colorectal cancer	857	10.4	Liver and gallbladder cancer	62	5.0
Lung cancer	749	9.1	Pancreas cancer	48	3.9
Liver and gallbladder cancer	693	8.4	Hematopoietic tumor	42	3.4
prostate cancer	376	4.6	Kidney cancer	32	2.6
Head and neck cancer	299	3.6	Thyroid gland cancer	25	2.0
Kidney cancer	279	3.4	Esophageal cancer	20	1.6
Pancreas cancer	253	3.1	Breast cancer	14	1.1
Esophageal cancer	194	2.4	Small intestine cancer	7	0.6
Thyroid gland cancer	160	1.9	Gynecologic cancer	6	0.5
Brain tumor	153	1.9	Prostate cancer	6	0.5
Musculoskeletal cancer	74	0.9	Mediastinal cancer	6	0.5
Small intestine cancer	59	0.7	Brain tumor	2	0.2
Mediastinal cancer	54	0.7	Musculoskeletal cancer	1	0.1
Skin cancer	40	0.5	Other	1	0.1
Other	31	0.4	Skin cancer	0	0
Malignant eye tumor	20	0.2	Malignant eye tumor	0	0
Male genital cancer	10	0.1	Male genital cancer	0	0
Malignant cardiac tumor	4	0.0	Malignant cardiac tumor	0	0
